# Effects of bacteriophages on gut microbiome functionality

**DOI:** 10.1080/19490976.2025.2481178

**Published:** 2025-03-31

**Authors:** Elena Kurilovich, Naama Geva-Zatorsky

**Affiliations:** a Department of Cell Biology and Cancer Science, Rappaport Technion Integrated Cancer Center (RTICC), Rappaport Faculty of Medicine, Technion – Israel Institute of Technology, Haifa, Israel; b Humans and the Microbiome program, CIFAR, Toronto, ON, Canada

**Keywords:** Gut microbiome, gut phageome, bacteriophages, bacterial functionality

## Abstract

The gut microbiome, composed of bacteria, fungi, and viruses, plays a crucial role in maintaining the delicate balance of human health. Emerging evidence suggests that microbiome disruptions can have far-reaching implications, ranging from the development of inflammatory diseases and cancer to metabolic disorders. Bacteriophages, or “phages”, are viruses that specifically infect bacterial cells, and their interactions with the gut microbiome are receiving increased attention. Despite the recently revived interest in the gut phageome, it is still considered the “dark matter” of the gut, with more than 80% of viral genomes remaining uncharacterized. Today, research is focused on understanding the mechanisms by which phages influence the gut microbiota and their potential applications. Bacteriophages may regulate the relative abundance of bacterial communities, affect bacterial functions in various ways, and modulate mammalian host immunity. This review explores how phages can regulate bacterial functionality, particularly in gut commensals and pathogens, emphasizing their role in gut health and disease.

## Introduction

Phages represent the vast majority of the human gut virome, composed of phages and eukaryotic viruses,^1^ with an abundance almost equal to that of bacteria.^2^ The interactions between phages and their bacterial hosts shape bacterial communities and influence their functionality. In the human gut microbiome, phages significantly contribute to the regulation of bacterial populations, promote genetic diversity, and drive the evolution of bacterial populations.^3,4^ They help spread beneficial traits, such as metabolic capabilities, and increase the adaptability and resilience of bacterial communities.^5–9^ On the other hand, phages can influence bacterial pathogenicity by directly targeting pathogenic bacteria, modulating the expression of virulence factors, or providing antibiotic resistance.^10,11^ Understanding the complex dynamics between phages and bacteria in the gut is essential for advancing our knowledge of microbiome ecology. Moreover, phages now represent a promising alternative to traditional antibiotics, especially in the fight against antibiotic-resistant bacteria. Insights into phage functionality can aid the development of innovative strategies for managing gut-related diseases.

This review focuses on the versatile roles of phages in regulating the functionality of gut commensals and pathogens, including the interplay with bacterial phase variation mechanisms. It also provides a comprehensive summary of the current findings on the impact of phages on gut health and their interactions with the mammalian host immune system. Furthermore, we explore the diversity of phage lifestyles and their prevalence in the gut and provide an overview of the most promising applications of phages in treating gut disorders and future research perspectives.

## Gut phageome

The gut phageome is increasingly recognized for its unique role in human health and disease. Despite the general assumption of phages as parasites, the relationship between bacterial and phage communities in a healthy gut environment is better described as predominantly mutualistic.^7^ Bacteria and phage communities in the human gut can co-exist for an extended period, remaining relatively stable (over 2 years).^6,12,13^ Moreover, phage and bacteria composition and their densities along the mammalian gastrointestinal tract (GIT) are positively correlated, measuring the lowest in the small intestine and the highest in the colon.^14^

The existence of a common core phageome was initially suggested and considered to be important for the stability of a healthy human microbiome.^15^ However, later studies^16,17^ demonstrated that the phageome is more person-specific, varying significantly across populations and age groups, and is mainly dependent on the individual microbiome.^18^ Given the high impact of diet on the gut microbiota,^19,20^ one could also expect considerable differences in phageome composition in populations consuming different diets. Another interpersonal phageome diversity factor is rapid phage evolution inside the gut.^13^ This process is driven by the dynamic interactions between phages, their bacterial hosts, and the mammalian immune system. As phages replicate within the gut complex ecosystem, they encounter a variety of selective pressures, such as nutrient and host limitations, competition with other phages, or bacterial anti-phage mechanisms, such as CRISPR-Cas or phase variation that lead to the emergence of new phage variants.^13^ These variants may possess expanded bacterial host ranges,^21^ improved replication efficiency, or increased resistance to bacterial defense mechanisms.

There are several ways bacteriophages could be classified. First, they can be divided based on their genetic material into four main types: double-stranded DNA (dsDNA), single-stranded DNA (ssDNA), double-stranded RNA (dsRNA), and single-stranded RNA (ssRNA) phages, though RNA phages appear to be rare in the human gut.^22^ Additionally, phages are divided by their morphology into tailed phages, which include myoviruses, siphoviruses, and podoviruses, which differ by tail size and structure, and tailless phages, which include filamentous, polyhedral, and pleomorphic forms. However, classification based on genetic material or morphology does not well reflect the phylogenetic relationships between phages, and the current classification accepted by the International Committee on Taxonomy of Viruses^23^ is based on genome similarity and phylogenetic analysis. The majority of gut phages belong to the class *Caudoviricetes* (dsDNA tailed phages) and the family *Microviridae* (small tailless ssDNA phages).^12^ The most prevalent and abundant phage group found in the gut is the order *Crassvirales* (crAss-like phages),^24^ tailed phages that infect bacteria of the phylum *Bacteroidota* (former *Bacteroidetes*). Metagenomic studies estimate them to be present in more than 70% of the global human population, with abundances reaching 99% in some individuals.^25^ Despite difficulties in gut phage culturing, the analysis of bacterial CRISPR arrays and prophage sequences in gut genomes demonstrated that different *Crassvirales* groups are associated with various *Bacteroidota* genera.^26,27^ Accordingly, the relative abundances of the *Crassvirales* genera are largely dissimilar^25,28^ between urbanized populations with Western diets, typically rich in *Bacteroides*, and populations with a high-fiber diet, such as the Hadza hunter-gatherers and other rural communities, where *Prevotella* species are more prevalent.^29–31^ A recent study that used a novel single-cell sequencing approach revealed a strong *in vivo* phage-host association between uncultured prototypical crAss (p-crAss) phage, the first *Crassvirales* phage discovered from the human viral metagenome analysis, and *Bacteroides vulgatus*.^32^ Additionally, a few crAss-like phages were successfully isolated which infect other *Bacteroides* species.^33–35^

In summary, the gut phageome is highly diverse and individual-specific, with more research required to further explore its diversity and underlying mechanisms.

### Phage lifestyle diversity

**Figure 1.**
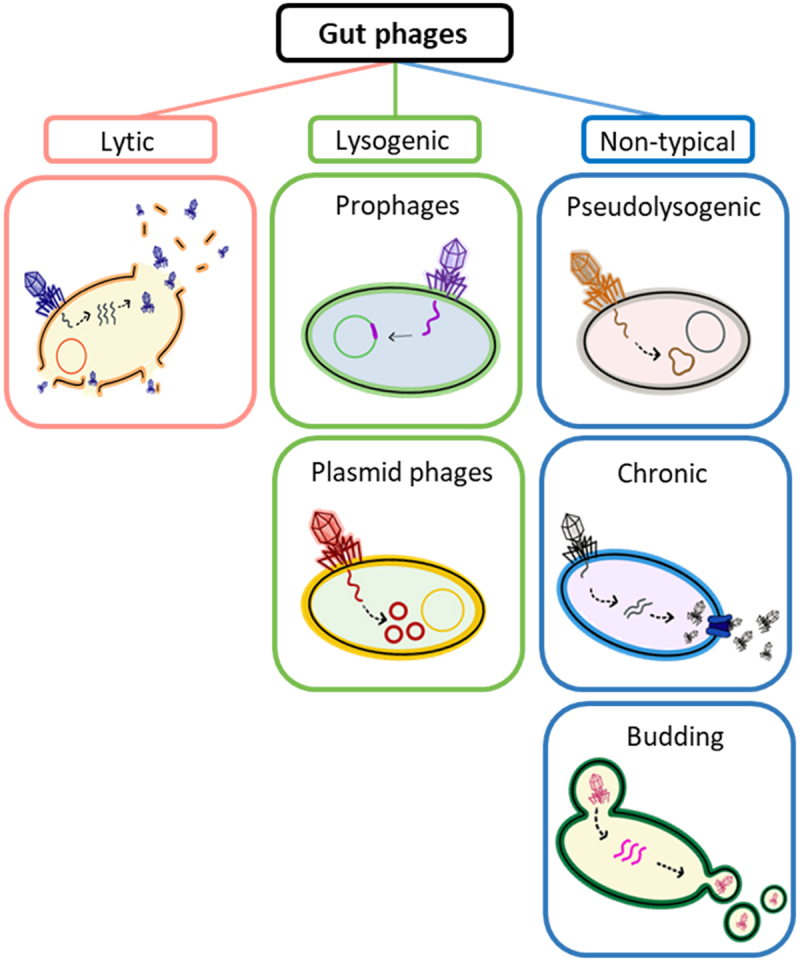
Classification of bacteriophage lifestyles.

Bacteriophages have two main lifestyles: lytic (virulent) and lysogenic (temperate) (Figure 1). Lytic phages use the resources of the bacterial host to proliferate and cause bacterial death upon progeny release. Lysogenic phages can integrate into the bacterial genome (forming prophages), comprising up to 20% of the bacterial genome,^36^ though the recent studies estimated the average content of prophages in the human gut bacterial genomes to be less than 5%.^37,38^ Some prophages persist as an extrachromosomal element, replicating alongside the bacterial genome (plasmid phages).^36^ Lysogenic phages get activated due to various environmental factors (described below), including temperature, oxidative stress, chemicals, or pH,^39^ which induce a switch to the lytic cycle.

Lytic phages are crucial in shaping the gut microbiota mainly by infecting and lysing specific bacterial hosts. Lysogenic phages, besides controlling bacterial populations, largely affect microbiome functionality by providing new traits, and their activity was strongly associated with microbiome-related diseases.^40–42^ Lysogenic phages comprise an important part of the gut phageome, estimated to be present in half of the gut bacteria.^36^ The impact of lysogenic and lytic phages on gut microbiota functionality is discussed in detail in the following chapters of the review.

Besides these two common lifestyles, some phages persist in bacteria as pseudolysogens,^43,44^ remaining as a non-replicating and non-integrating plasmid inside bacteria. Such a state appears to be transient and is caused by environmental conditions unfavorable for the bacteria, such as bacterial starvation. Pseudolysogenic phages may switch to a lytic or lysogenic cycle upon environmental change. Pseudolysogeny was demonstrated *in vitro* for several *Escherichia coli* and *Pseudomonas aeruginosa* phages,^45,46^ but due to their hidden character and culturing difficulties, gut pseudolysogens *in vivo* and their possible ecological role in the gut are largely understudied. Continuous culture experiments suggested that pseudolysogeny contributes to better phage survival in conditions with limited resources,^44,46^ which can occur in the gut. *In vitro* studies also proposed pseudolysogenic phages to support the accumulation of mutations conferring phage resistance in bacteria.^47^

“Chronic” life cycle is common for filamentous phages of the *Inovirus* genus and allows a long-lasting bacteria-phage co-existence. Chronic phages infect mainly Gram-negative bacteria, particularly several well-known gut pathogens, such as *Escherichia coli*, *Pseudomonas aeruginosa*, *Vibrio cholerae*, and *Salmonella enterica*.^48^ Some chronic phages can be incorporated into the genome and exist in a prophage form, but unlike lysogenic phages, they are not activated and cannot become lytic.^49,50^ The inserted filamentous phage may benefit the bacterial host by providing new functions and virulence factors,^51^ similar to lysogenic phages. However, during the chronic cycle, phages, including those incorporated into the bacterial genome, continuously release new phage particles from the bacteria without cell lysis. The size of the filamentous phages underlies its assembly on the bacterial membrane, followed by the active secretin-dependent release.^52^

The unique egress mechanism described for *Plasmaviridae* phages is called budding. The dsDNA of these phages lack capsids and are surrounded by the bacteria-derived lipid membrane. New phage particles of *Plasmaviridae* are released from the cell using liposomes in a process called budding.^53^ Though budding is performed without lysis, the continuous phage particle release may lead to the bacterial death.^54^ The prevalence and functionality of *Plasmaviridae* in the human gut are poorly studied, as well as the role of their host bacteria in the gut.^55,56^ However, given their membrane fusion infection mechanism, a broad host range could be proposed for this phage group.^57^

Lastly, another type of phage-bacteria interaction, termed “carrier state”, comprises a stable long-term co-existence of bacteria and phage in culture. In a carrier strain, most bacteria are resistant, while a small proportion remains susceptible to the phage, thus supporting its propagation.^58^ In this case, bacteria are not lysogenic, and phages could be eliminated from the mixture by plating or treatment with an anti-phage serum. Carrier state infection was demonstrated *in vitro* for the human opportunistic pathogen *Pseudomonas aeruginosa* ^59^ and proposed to promote bacterial evolution. For *Campylobacter jejuni* ,^60^ causing diarrheal disease, the carrier state infection was demonstrated by *in vitro* experiments to aid stress tolerance and survival of bacteria outside the gut environment.^60,61^

## Gut prophages

The human gut microbiome increases in complexity from infancy to adulthood.^62^ Prophages were shown to be more prevalent and diverse in an infant’s gut than in adults, forming the major part of the early-life virome.^63^ Later, as the microbiome becomes more diverse and rich, the gut is colonized by more lytic phages and eukaryotic viruses.^63^ Therefore, prophages play an active role in establishing the gut microbiota^17^ while remaining widespread in adults.

Prophages may benefit their host bacteria in multiple ways. First, they may provide bacteria with auxiliary metabolic genes (AMGs),^64,65^ causing lysogen conversion^66^ and increasing bacterial fitness. This ability becomes particularly important for pathogenic bacteria, where prophages can encode various virulence factors and antibiotic-resistance genes.^67–69^ A common feature of prophages, beneficial to the bacterial host and the prophage itself, is the ability to suppress secondary infections by the same or a related phage. This way, prophages ensure their exclusive access to bacterial resources and protect their genetic integrity. The first mechanism, termed “superinfection exclusion” (SIE), was demonstrated for a wide range of phages, primarily preventing secondary infections at the adsorption or DNA injection stages.^70,71^ Another mechanism, superinfection immunity (Sii), inhibits the secondary infecting phage DNA replication and transcription by the same inhibitory proteins which ensure the lysogenic state of the primary phage.^72–74^

Moreover, prophages actively participate in transduction, a process of phage-mediated genetic exchange between bacterial cells. This can occur through specialized transduction,^75,76^ where prophages excise imprecisely and package the nearby bacterial DNA, or through lateral transduction,^77^ where prophages replicate while still integrated due to the late excision, leading to a highly frequent packaging of large adjacent bacterial DNA fragments. Once activated, some prophages are able to mediate generalized transduction,^78,79^ packaging bacterial DNA fragments into the capsid. Upon infection of another cell, the transferred DNA may be inserted into the new bacterial genome by homologous recombination. Transduction greatly contributes to genetic diversity and evolution within bacterial populations^80^ and was strongly suggested to be widespread in murine^81^ and human gut.^82^

Although prophage insertion might benefit the bacterial host (increased fitness, superinfection inhibition, advantageous gene transfer), there can also be negative consequences (gene disruption, induced cell lysis), resulting in a trade-off.^8,83^ Lysogeny is associated with substantial fitness costs due to the need to carry additional genetic material and fatal risks after prophage induction.

Despite the high prevalence of lysogenic phages in the human gut, little is known about their functional and ecological impact on the gut microbiome. The following chapter describes the role of prophages in the fitness and functionality of both gut commensals and pathogens.

### Prophages influence gut commensal functionality

**Figure 2.**
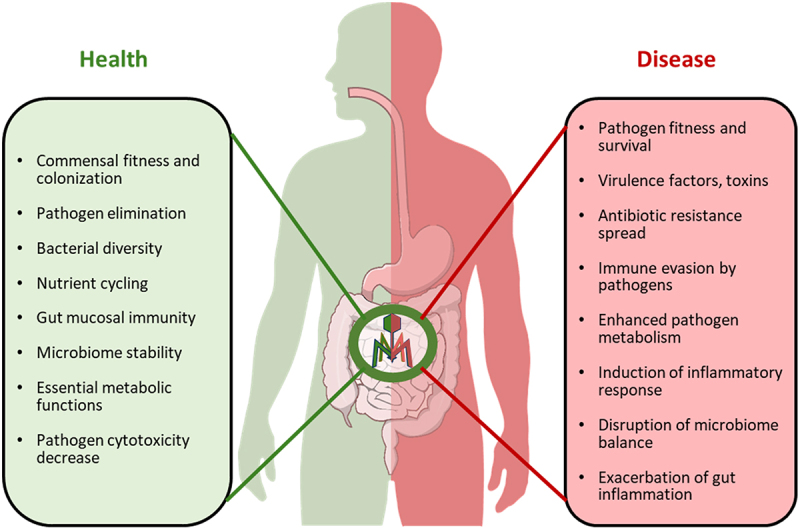
Gut phages affect functionality of gut commensals and pathogens, contributing to health and disease.

The data obtained so far on the role of bacteriophages in healthy gut microbiome functionality is limited. However, the research points to the importance of phages in gut microbiota metabolism and fitness (Figure 2).^9^

The metagenomic analysis of 124 individual European gut samples revealed that prophages perform up to 5% of the known core functions of the human gut microbiota, taking part in nutrient cycling and population stability. Metagenomic analysis of VLPs (virus-like particles) from 32 fecal samples from four pairs of adult female monozygotic twins and their mothers at three time points revealed that many gut phage-encoded proteins provide essential metabolic functions to their microbial hosts, adapting them to anaerobic conditions.^6^ These proteins are involved in transcriptional regulation and synthesis of nucleotides, essential metabolites, amino acids, and peptidoglycans, highlighting the role of prophages in gut microbiome fitness.

In a more recent study, single-microbe RNA (smRNA) sequencing of the bacterial transcriptome in four fecal samples of healthy adults showed that prophage-encoded functional genes (at least those that could be annotated) commonly take part in crucial functional pathways, such as arginine and tryptophan metabolism and the bacterial stress response.^84^

One of the examples of prophages affecting the metabolism of a gut commensal is the BV01 prophage of *Bacteroides vulgatus*, which affects bile salt hydrolase (BSH) activity.^85^ BV01 prophage disrupts the promoter region of *tspO* (tryptophan-rich sensory protein/translocator protein). As a result, it represses *tspO*-dependent transcription of the BSH gene, which is responsible for the deconjugation and amidation of bile acids. Bile acids conjugate with taurine or glycine to produce bile salts in the liver and are secreted to the small intestine, facilitating fat absorption. Bile salt hydrolysis is performed by BSHs from the gut microbiota,^86,87^ forming free unconjugated bile acids, secondary bacterial bile acids, and subsequently bacterial bile acid amidates (BBAAs). Alterations in BSH activity are largely associated with metabolic syndrome and obesity.^86^ BSH was also shown to affect the growth of *Clostridium difficile* in mice intestines *in vivo* and in human fecal samples *ex vivo* and was demonstrated to influence local viral susceptibility in the murine gut.^88^ Though the proportion of BV01 lysogens was shown to be generally low in individuals, it is common in populations, suggesting a frequency-dependent regulation mechanism.^85^

Interestingly, conjugated bile acids were shown to induce the production of Bxa by the *Bacteroides stercoris* prophage.^89^ Bxa belongs to the bacterial ADP-ribosyltransferases (ADPRTs), which are pathogenic toxins capable of changing the metabolism of the gut epithelium. *B. stercoris* phage-encoded Bxa affects the gut epithelial cytoskeleton and causes inosine secretion, which bacteria use as a carbon source.^89^ This function makes Bxa a prophage-encoded fitness factor, which provides benefits in bacterial adherence and colonization in the gut. Moreover, various prophage-encoded ADPRTs were found to be widespread among common gut commensals, including *Bacteroidetes*, *Firmicutes*, *Actinobacteria*, and other phyla.^89^

Analysis of infant gut prophages showed they are involved in the dTDP-L-rhamnose and menaquinone (vitamin K) biosynthesis pathways of bacteria.^17^ The dTDP-L-rhamnose pathway is involved in the biosynthesis of the O antigen of LPS, which was shown to be important for phage susceptibility.^90–92^ Some phages are known to encode LPS biosynthesis proteins using them for the SIE mechanism. The O antigen of LPS influences bacterial interactions with the mammalian host immune response by helping bacteria to evade the complement system^93–96^ and represents a critical virulence factor in the case of pathogens, such as *Yersinia enterocolitica* and *P. aeruginosa* .^97^

Prophages may also increase their bacterial host’s fitness relative to non-infected bacteria. In *Lactobacillus reuteri*, active prophages are widely present throughout different strains and hosts. *In vivo* mice experiments showed that prophages provide an advantage to lysogens by outcompeting sensitive strains in a gut.^98^ Competition experiments with *Enterococcus faecalis* infected or not infected with the ϕV1/7 phage demonstrated that the infected strain was able to produce new phage particles and outcompeted the uninfected strain, both *in vitro* and *in vivo*.^99^ Moreover, in a study of the intra-personal evolution of *Bacteroides fragilis*, a prophage was identified that provided a competitive advantage to one of the lineages through prophage-mediated killing of the prophage-lacking bacteria.^5^ Interestingly, a years-long coexistence of the two lineages was observed, suggesting a balancing mechanism supporting population diversity. These results in two of the main gut commensals emphasize that gut prophages might play an important role in bacterial colonization of the gut, thus influencing microbiome composition.

Often, the insertion of a prophage into the host genome disrupts bacterial genes or regulatory sequences. However, such disruptions may be reversible and act as phage regulatory switches (phage-RS), which can control gene expression according to environmental conditions.^100^ An example of this mechanism implemented in a gut commensal is the Skin (*sigK*-intervening) DNA element. *Bacillus subtilis* implements this phage-RS to regulate sporulation, a process crucial for bacterial adaptation and survival in the gut environment. The Skin element comprises a cryptic prophage that separates the *sigK* gene and can reversibly be excised from the genome, thus restoring the gene and controlling the late sporulation stage.^101^ Notably, the Skin element cannot generate mature phages, thus resembling a non-infective phage-RS. A similar mechanism was also reported for the gut pathogen *Clostridium difficile*.^102^ Another sporulation-related phage-RS of *B. subtilis*, SPβ, is inserted into the *spsM* gene, crucial for the adhesive and hydrophilic properties of the spore envelope.^103^ Once SPβ is excised from the genome during sporulation, the functional *spsM* is restored. Unlike the Skin element, SPβ is an active prophage, which can maintain the ability to get activated in response to DNA damage and propagate.^104^ *In vivo* research on mice^105^ and *in vitro* experiments on chicken^106^ and human^107^ *B. subtilis* isolates suggest sporulation is an important mechanism ensuring the survival and adaptation of *B. subtilis* to the gut environment.^105,106^

In summary, prophages help gut commensals to adapt to the gut environment, impact metabolic processes, and provide competitive advantages, thus influencing the composition and functionality of the gut bacterial community.

### Prophages in gut pathogens

Prophages greatly contribute to the complexity and adaptability of bacterial pathogens, enhancing their virulence, antimicrobial resistance, and immune evasion (Figure 2). Some of the most relevant examples of such interactions are discussed below.

A prophage-encoded *sopE* gene was shown to enhance the growth of *Salmonella Typhimurium*, which can cause severe enteric infections. *SopE* was shown to enable nitrate respiration in the inflamed murine gut.^11^ The gene enables an increase in the production of inducible nitric oxide synthase (iNOS) and, subsequently, nitrate, an energetically valuable electron acceptor, suppressing the use of less efficient electron acceptors like tetrathionate, thus enhancing *Salmonella*’s fitness in the gut. This mechanism is also relevant for other *Enterobacteriaceae* pathogens, highlighting the role of bacteriophage-mediated horizontal gene transfer (HGT) in pathogen fitness and evolution.^11^

A study of lambda prophage in *E. coli* demonstrated that the prophage-encoded cI protein, a factor protecting bacteria from infection by other phages and regulating the prophage’s expression, also directly inhibits the *pckA* gene of bacteria.^10^ The down-regulation of *pckA* affects gluconeogenesis and lowers bacterial growth rates in energy-poor environments. Moreover, the *pckA* regulatory region contains multiple binding sites for other lambdoid phage-encoded factors, pointing to a strong selection for the described regulatory mechanism. Though the exact explanation is still missing, a lowered growth rate is suggested to increase the chance for the lysogens to survive in the gut environment and evade the immune system.^10^

Another study revealed a crucial role of prophages in the release of Colicin Ib (ColIb) by *Salmonella enterica* serovar *Typhimurium* .^108^ An *Enterobacteriaceae*-specific bacteriocin Collb confers a strong benefit to *S. Typhimurium* over competing Collb-sensitive *E. coli* in the inflamed murine gut.^109^ The lysis of *S. Typhimurium* by the activated temperate lambdoid phages *in vitro* causes ColIb release into the environment, enhancing the advantage of *S. Typhimurium* population over *E. coli*.^108^ This interaction highlights a novel mechanism of temperate phages in promoting pathogen fitness.

Shiga toxins, produced by pathogenic *Shigella dysenteriae* and some *E. coli* strains, are among the most potent toxins. In *E.coli* O157:H7, Shiga toxins Stx1 and Stx2 are encoded on two lambda-like prophages, Sp5 and Sp15, respectively.^67,110^ The toxins are produced upon prophage induction, which leads to severe diseases, including hemorrhagic colitis and life-threatening hemolytic uremic syndrome (HUS).^111,112^ In *S. dysenteriae* serotype 1, Stx is also associated with lambdoid phage genes, though it is expressed from the bacterial chromosome and not encoded by the active prophage.^113^ Stx is one of the main virulence factors of *S. dysenteriae*, being responsible for the disease severity.^114,115^

Pathogenic strains of *V. chlolerae* cause severe cholera infections, accompanied by serious diarrhea, dehydration, and electrolyte imbalance, often causing death.^116^ The symptoms are mainly caused by cholera toxin (CT) encoded by the *CTXϕ* filamentous bacteriophage irreversibly integrated into the genome.^51^ *CTXϕ* infects *V. chlolerae* using the toxin-coregulated pilus (TCP) as a receptor, which is also essential for the gut epithelium colonization. Unlike typical prophages, *CTXϕ* is able to replicate without getting excised from the genome, producing new phage particles while remaining integrated in a bacterial chromosome.^117^

In pathogenic clostridia, virulence factors are often associated with phages, though their presence and potential to enhance virulence can vary between strains. Phages persisting in some pathogenic *C. botulinum* strains carry the botulinum neurotoxin (BoNTs) genes, which are responsible for the deadly botulism disease, causing flaccid paralysis.^118–121^ These phages were found to persist as unstable plasmids, resembling pseudolysogens.^120–122^ The primary virulence factor of *C. novyi*, a deadly pathogen causing a wide range of serious conditions such as soft tissue infections, is α-toxin, which was also shown to be encoded by a plasmid phage.^123,124^ *C. difficile* causes dangerous colon infections leading to diarrhea and colitis.^125^ Though its toxin genes are commonly considered chromosomal, data has been accumulating showing a crucial role of prophages in *C. difficile* toxicity regulation and spread. The main *C. difficile* toxins, TcdA and TcdB, are encoded on a PaLoc locus comprising a part of an ancient prophage.^126^ Some prophages, such as *ϕCD119*, were shown to activate PaLoc by expressing its transcription regulators.^68^ Furthermore, the *C. difficile* binary toxin locus CdtLoc, typically encoded on the bacterial chromosome, was also found on *phiSemix9P1* prophage, highlighting the role of prophages in spreading toxigenicity among bacteria.^69^ Some prophage-encoded toxins were found in several isolates of *C. perfringens* ,^127^ a bacteria causing a variety of systemic and gastrointestinal diseases in human or animals.^128^ Prophages were also proposed to participate in *C. perfringens* sporulation regulation,^129,130^ crucial for the bacterial colonization and pathogenicity.^131^ Still, the role of these prophages in *C. perfringens* pathogenicity is to be further studied.

In addition to aiding pathogen virulence, bacteriophages might also counteract virulence-related genes. For example, the temperate PHB09 phage integrates inside the pilin gene of *Bordetella bronchiseptica*, a common respiratory tract pathogen, which significantly decreases bacterial virulence.^132^ This effect is most likely explained by abolished pilin expression. Pilin proteins form pili, which are suggested to play a role in bacterial adhesion^133^ and signal transduction in pathogens, thus comprising an important virulent factor.

Overall, prophages greatly influence gut pathogen survival, virulence, and fitness. However, there are indications that they can also reduce the pathogenicity of bacteria by disrupting virulence-related genes. This dual role underscores the complex impact of prophages on bacterial evolution and pathogenicity.

The impact of gut prophages on microbiome functionality is highly extensive and employs various mechanisms. Their understanding can help to develop new approaches to manage gut infections and maintain a healthy microbiome.

### Prophage activation

Lysogen induction is the process of dormant phage activation. Upon induction, prophage starts expressing itself and produces lytic phage particles that can infect other cells. The activation factors greatly vary across different phages. In the gut environment, lysogens encounter various environmental stressors able to cause their induction,^39,134^ such as pH changes,^135^ oxidative stress,^136^ temperature or chemicals, including antibiotics,^137^ and other factors described below. Some lysogens are activated more frequently in the murine gut than *in vitro*, mainly due to the bacterial SOS response,^138^ which may be caused by diet^139^ or other mammalian host factors.^39,140^ Still, studies to date demonstrate that only a minor fraction of gut prophages is inducible.^141–143^

Prophage activation has been associated with affected gut microbiome composition and inflammation in humans and mice, particularly in Inflammatory bowel disease (IBD).^40,42^ Gut inflammation causes strong lysogen activation through the reactive oxygen species (ROS)- or NO-induced SOS response. The resulting products of increased bacterial lysis could further induce a pro-inflammatory response, thus aggravating the disease.^41^ Metagenomic analysis of microbiota composition and viromes derived from healthy and IBD patients revealed increased amounts of *Firmicutes*-infecting temperate phages in IBD, while *Firmicutes* abundances were decreased,^41^ which could be explained by prophage activation linked to the disease. Therefore, the process of prophage induction is highly relevant for gut microbiota-related research.

Prophage induction can be controlled by the bacterial metabolic state.^39^ For instance, the lysogeny of T1 prophage in *E. coli* was shown to be regulated *in vitro* by the bacterial cAMP levels.^144^ The production of specific metabolites in the gut by the lysogenic bacteria might also lead to prophage induction. For instance, *E. coli*-produced toxin colibactin was shown to induce prophages through the SOS response activation in this and other neighboring bacteria *in vitro*.^145^ In the human commensal *Lactobacillus reuteri*, prophages are activated *in vitro* by short-chain fatty acids resulting from fructose metabolism.^139^ Quorum-sensing signals were also demonstrated to induce prophages *in vitro*, not only in pathogenic *V. cholerae* and *E. coli*^144,146^ but in commensal *Enterococcus faecalis* as well.^147^ However, this observation has yet to be proven by *in vivo* experiments.

Gut mucus density gradually decreases from the epithelium to the lumen while the bacterial load increases. A modeling study^148^ suggested spatial mucus structure influences the replication strategy of gut phages, with lysogeny dominating at the top layers and lysis favored closer to the epithelium, in good agreement with the Piggyback-the-Winner model, shortly explained as “more microbes, fewer viruses”.^149^ This observation suggests that high bacterial densities and growth rates support the temperate phage lifestyle, while the lytic pathway is predominated at lower bacterial densities. Such spatiality may greatly contribute to gut health by protecting the mucus from pathogen invasion and supporting commensal colonization, providing it with fitness benefits.^148^

## Gut lytic phages

Lytic phages can significantly influence the composition and function of the gut microbial community by specifically lysing their host bacteria. Notably, lytic phages not only regulate their host bacteria population but also effect other species and change the microbiota metabolome.^150^ A study that used gnotobiotic mice harboring nine commensal bacterial species demonstrated the close-knit inter-bacterial interactions in the gut, such as the elimination of a particular species by its phage causing a cascading effect on others. For instance, the administration of *E. coli*-targeting T4 phage and *Clostridium sporogenes*-targeting F1 phage to the mice caused observable changes in the abundance of *Akkermansia muciniphila* and *B. fragilis*, while the overall bacterial load remained stable. Despite the known metabolic redundancy of different bacteria in the gut, the metabolites uniquely associated with particular species were also affected by phage predation. For example, treatment with *C. sporogenes*-targeting phages reduced the levels of the neurotransmitter tryptamine, which is uniquely associated with *C. sporogenes* and affects gastric motility. Unlike the broad influence of antibiotics on the gut metabolome, the metabolic effects of phage treatments are considered to be much more precise, allowing a highly targeted therapeutic approach. A study that used anaerobically cultivated human intestinal microflora demonstrated high specificity of phage treatment against *Salmonella* infection, compared to antibiotic treatment.^151^ 16S DNA and RNA sequencing revealed that while antibiotics significantly altered the commensal composition, phage treatment preserved the community. In another work, a lake-derived bacterial community was infected with *Flavobacterium columnare* and subsequently treated with either the *Flavobacterium*-targeting bacteriophage or antibiotic.^152^ Flow cytometry analysis and 16S rRNA gene sequencing showed the drastic effects of antibiotic treatment on community density and diversity, as opposed to the minor effects of the phage. Therefore, lytic phages are being widely explored as alternatives to antibiotics for treating bacterial infections, including those in the gut.

Lytic gut phages may also drive the evolution of gut bacterial communities, leading to a wide range of anti-phage mechanisms and various mutations.^3^ The acquired mutations, which protect bacteria from phage predation, may also affect bacterial metabolic properties. Therefore, phage predation is important in shaping bacterial diversity in the gut.^4^ Lytic phages, as well as prophages, may also participate in the HGT by generalized transduction process,^153–157^ where bacterial DNA fragments are erroneously packaged together with phage DNA or instead of it and then transferred to another host during the next infection cycle. Though transduction by lytic phages is much less efficient compared to lysogenic phages, it is still considered to contribute significantly to genetic diversity in bacterial populations.

Moreover, the phage-caused lysis of bacterial cells releases nutrients into the gut environment, which can be utilized by other bacteria, contributing to metabolic activity within the microbiota. The great role of lytic phages in nutrient cycling is well recognized for the ocean^158,159^ and soil^160^ environment and could be proposed for the gut microbiome as well.

Another intriguing way lytic phages might influence bacterial functionality is by affecting phase variable genes. The known examples of such interactions are described in the next chapter.

## Phages and phase variation in gut bacteria

Phase variation is a widespread adaptation mechanism utilized by gut bacteria to mediate a rapid and reversible control of gene expression. Phase variation mechanisms include site-specific recombination, DNA methylation, and slipped strand mispairing.^161^ It is common for both commensals and pathogens and has multiple functions. Besides regulation of bacterial virulence and persistence,^162,163^ it also interferes with phage infection. There is evidence proving that phages might also affect the phase variable genes by various mechanisms, thus modulating the functionality of bacteria. Such interactions were revealed for the several well-known gut commensals, described below in more details.

Some phages use bacterial polysaccharides as receptors, and phase variation of the polysaccharides genes can cause a transient and reversible resistance, with a part of the population remaining sensitive. In *Bacteroides thetaiotaomicron*, capsular polysaccharides (CPS) controlled by phase-variable mechanisms participate in phage evasion.^34,90^ Researchers found that the expression of particular CPS variants is selected under phage predation, enabling survival. Therefore, phase variable genes regulate phage susceptibility, providing a transient phage resistance in the population. CPS in *Bacteroides intestinalis* are also phase-variable and may switch between different variants. Similarly, to the previous example, this variation was demonstrated to allow some bacterial cells to become temporarily resistant to ΦcrAss001 phage infection, while others remained sensitive.^164^ Moreover, ΦcrAss001 demonstrated a delayed burst *in vitro*, allowing *B. intestinalis* to live and function for a longer period before lysis. Such a bacteria-phage relationship follows a Piggyback-the-Winner model, supporting a continuous and stable co-existence of the phage and its host in a gut.

A unique example of prophage-mediated phase variation regulation was discovered in *Clostridium difficile*. The ϕCD38–2 prophage changed the abundance of the bacteria expressing the phase-variable cell wall protein CwpV from 5% to 95%.^165^ The gene was shown to be upregulated ∼20-fold in the lysogen, but the mechanism of this regulation remains unclear. The ON/OFF switch is mediated by the bacteria-encoded recombinase RecV and the prophage is suggested to interfere with RecV or another bacterial factor responsible for the switch. Later, CwpV was demonstrated to be highly protective against phage infection (DNA injection). Thus, the explored mechanism comprises a variant of the superinfection exclusion^166^ encoded by the bacteria, while the prophage role in it is to be further investigated.

An exceptional case of phages employing phase variation was demonstrated^167^ for *Fletchervirus* phages, which infect *Campylobacter jejuni*, a well-known gut pathogen causing diarrhea. *Campylobacter* posess hypermutable polyG tracts in various genes participating in the surface molecule synthesis. *Fletchervirus* phages were found to use similar polyG tracts to create phenotypic diversity in their receptor-binding proteins to evade bacterial resistance.

In a recent study^168^ focused on connections between gut inflammation and phase variation in bacteria, a novel role of bacteriophages was explored. The polysaccharide A (PSA) promoter OFF orientation in *B. fragilis* was found to be not only associated with IBD in both humans and mice, but also with reduced colonic Tregs in mice and increased *B. fragilis*-associated bacteriophage levels in humans. The experiments on the mice model demonstrated that the infection with the lytic phage Barc2635 caused the PSA promoter to switch from ON to OFF orientation, resulting in a subsequent drop in Tregs. Interestingly, the phase variation state did not influence phage infectivity, suggesting a regulation distinct from the one previously demonstrated for *B. thetaiotaomicron* and *B. intestinalis*. The mechanisms of the relationships between phages, phase variation, and inflammation are yet to be studied and hold great potential for the development of novel diagnostics and therapy.

## Gut phages modulate host immunity

Bacteria play an important role in regulating the mammalian immune responses. These interactions are highly relevant in both the healthy state and various disease conditions.^169,170^ However, evidence is accumulating showing that phages are also able to modulate mammalian immunity (Figure 2) by changing bacterial functionality and abundance.

A study which used human blood neutrophils and monocytes showed that specific phages were able to decrease bacteria-induced ROS production in phagocytes.^171^ A later study proved the observation using both LPS- and bacteria-induced polymorphonuclear leukocytes.^172^ The effect could be explained by the phage adhering to bacteria or LPS in particular, thus preventing their interaction with immune cells. ROS are crucial for antibacterial functions of phagocytes, but can cause tissue damage when produced excessively, which can be particularly relevant for the viral infections and sepsis.^173,174^ Thus, phages could be potentially implemented in treatment of inflammatory conditions and infections accompanied with oxidative stress.

A protective role of phages was also proposed by *in vitro* experiments, which showed that gut phages can adhere to mammalian mucus components. The immunoglobulin-like domains in phage capsids were demonstrated to attach to mucins, thus protecting the underlying epithelium from bacterial invasion.^175^ Though the relevance of the explored mechanism for the *in vivo* conditions is yet to be studied, it suggests an important role of phages in mucosal immunity.

Cross-infection experiments using human microbiota-associated mice and VLPs from ulcerative colitis (UC) and healthy patients demonstrated that fecal virome transplantation (FVT) from diseased donors increases DSS colitis severity.^176^ In a similar study,^177^ viral transfer from IBD (both ulcerative colitis and Crohn’s disease) or non-IBD patients to human-associated mice exacerbated inflammation or elicited an anti-inflammatory response, respectively. The results were further supported by *in vitro* experiments, where co-incubation of macrophages with IBD- or non-IBD-derived viromes led to corresponding pro- and anti-inflammatory responses. Besides bacteriophages, VLPs also contain eukaryotic viruses, thus making it challenging to confirm the distinct role of phages in observed effects. However, the altered phageome composition and phage-bacteria associations in IBD patients^178,179^ and mice^180^ point to a significant role of phages in modulating immune responses and contributing to the pathogenesis of IBD, thus providing a potential for novel IBD diagnostics and therapy.

Similarly, the analysis of the phageomes from individuals at risk for rheumatoid arthritis (RA) and healthy controls by metagenomics sequencing revealed significant differences in phage communities and their metabolic functions.^181^ Importantly, the AMGs involved in LPS biosynthesis and biofilm formation were found to be differentially present in RA and healthy samples, which can potentially impact the human immune response. The results propose that phages might be utilized in early diagnostics of RA, and their possible role in RA progression should be further studied.^181^

Moreover, the research data accumulating on direct interactions of phages with mammalian cells, both *in vivo* and *in vitro*, indicates that phages might also directly affect mammalian immune responses. Filamentous Pf phage, that chronically infects *P. aeruginosa*, was demonstrated to promote *P. aeruginosa* infection of wounds by directly interacting with immune cells and suppressing the phagocytosis of *P. aeruginosa*.^182^ Pf transcription inside the immune cells upon phage internalization caused TLR3-dependent response and affected TNF production, required for the bacterial infection clearance.

The effects of direct interactions of phages with immune cells could be more pronounced in patients with IBD, which is characterized by the damaged mucosal protective barrier and a burst of free bacteriophages in the gut. An extensive study that used both *in vitro* and *in vivo* methods demonstrated that phages can directly stimulate mammalian immune responses.^183^ Particularly, a continuous *E. coli* phage treatment of germ-free (GF) mice led to the CD4+ T cell expansion in the gut and an elevated number of IFN-γ producing T cells in Peyer patches. *In vitro*, dendritic cells incubated with various phages were shown to potently induce TLR9-dependent IFN-γ production in CD4+ T cells. Moreover, phage treatment was shown to activate both specific and nonspecific immune responses and exacerbate colitis in specific pathogen-free (SPF) mice which lacked the targeted bacteria, pointing to the direct effect of the phage. In another study, phages internalized by the lung and kidney epithelial cells did not cause TLR9 response *in vitro*. Instead, phage internalization activated AKT and inhibited CDK1 signaling pathway, resulting in increased cellular growth and metabolism.^184^

Several studies demonstrated that phages can be internalized *in vitro* by phagocytic cells such as macrophages and dendritic cells.^183^ The latter could translocate phages to systemic organs. While the dissemination of bacteria in blood and organs is extensively studied,^185–187^ there is a lack of research focusing on the presence of phages in different organs and its possible consequences. In a study of sarcoidosis patients, ~75% of diseased individuals harbored mycobacteriophages in their blood serum, while no phages were found in the blood of healthy individuals or tuberculosis patients.^188^ In contrast, another study measured phages in the circulating blood of both healthy people and those with Crohn’s disease with an equal frequency.^189^ Metagenomic analysis of domestic pigs and rhesus macaques demonstrated the natural presence of gut phages, mainly *Microviridae*, in parenchymal organs, such as lungs, liver, and spleen, pointing to the ability of healthy gut phages to penetrate the gut and reach other organs.^14^

## Phage applications

The rapidly growing problem of antibiotic resistance has led to a resurgence of interest in phage therapy. If used as a substitute or a supplement to antibiotics, it could greatly enhance our current capability to treat multidrug-resistant infections. In contrast to the broad and unspecific action of antibiotics, leading to multiple and long-lasting changes in the gut microbiota, phages can act specifically on particular strains or species. Despite the close inter-species relationships in the gut consortium, the implementation of targeted phage therapy could greatly reduce the risk of unwanted side effects.

Lytic phages and phage cocktails hold great potential for the treatment of bacterial infections, which has been demonstrated by multiple studies.^190–193^ One of the promising examples is a phage-based treatment of a *Staphylococcus aureus* infection, which is difficult to fight due to the rapid development of antibiotic resistance and its ability to persist for a long time inside phagocytic mammalian cells, including macrophages. These factors further complicate antibiotic therapy and lead to the spread of the infection. A study using mouse peritoneal macrophages demonstrated that MR-5 phages adsorbed onto *S. aureus* can significantly reduce the number of viable intracellular bacteria.^194^ MR-5 uses *S. aureus* as a vehicle to penetrate into the macrophages and lyse the intracellular bacteria, which makes the eradication of *S. aureus* more effective. Moreover, phages significantly reduced the bacterial cytotoxic effects on macrophages. The results were later supported by *in vivo* experiments on a murine air pouch model,^195^ showing that MR-5 alone and in combination with antibiotic linezolid is effective against *S. aureus* infection. Using combined phage-antibiotics therapy in the treatment of various multidrug-resistant infections was proposed as highly promising by many other studies.^196,197^ The combined approach is able to prevent the development of phage and antibiotic resistance^198–201^ and shows a higher efficiency due to the phage-antibiotic synergy.^202^ Recent studies also demonstrated that phage cocktails hold promise in modulation of Type II diabetes,^203^ nonalcoholic fatty liver disease,^204^ and *Salmonella* infections.^190,205^

IBD, characterized by an altered immune response, is widely associated in humans with colonization by several gut pathobionts, such as *Klebsiella pneumoniae*,^206^ which exacerbate the disease. A bacteriophage cocktail against *K. pneumoniae* was demonstrated to decrease gut inflammation in mice in the IBD model and proposed as a novel approach for the IBD treatment.^193^ The precision of phage therapy, in contrast to antibiotics, would maintain a gut microbiome balance, which is important for managing IBD.

In the case of *Clostridioides difficile* infection, phages, particularly *Caudoviricetes*, were demonstrated to play an important role in the efficiency of fecal microbiota transplantation (FMT) by many studies.^207,208^ Though the exact mechanisms remain unknown, phages could influence gut bacterial composition and functionality and disease elimination. Moreover, fecal virome transplantation (FVT), which uses filtered donor stool containing gut viruses and metabolites was shown to be effective in *C. difficile* treatment, causing long-term changes in bacterial and viral communities in patients.^209^ Experiments on mice models also demonstrated the potential of FVT in the treatment of metabolic disorders,^210,211^ dysbiosis,^212^ and necrotizing enterocolitis.^213^

Temperate phages could also be used in therapy, as an effective tool to modify the bacterial genome. They can be implemented to neutralize specific gut bacterial toxins and turn off other virulence factors. Hsu et al.^214^ demonstrated the use of the genetically engineered λ prophage to block Shiga toxin (Stx) production in *E. coli* both *in vitro* and *in vivo*. Stx is one of the most harmful prophage-encoded toxins expressed by enterohemorrhagic *E. coli*, and the approach could allow effective virulence neutralization. Prophages can also be used to resensitize pathogens to antibiotic treatment, either by introducing relevant genes^215^ or by antibiotic-induced lysogen induction.^216^ Moreover, prophage induction by specific dietary compounds was proposed as a way to modulate gut microbiome.^217,218^

An important role of gut microbiota was demonstrated in colorectal cancer (CRC). Particularly, *Fusobacterium nucleatum* was shown to contribute to immune-suppressive CRC microenvironment and tumor progression.^219–221^ A study that used a mouse CRC model demonstrated efficient elimination of *Fusobacterium nucleatum* via binding by specific M13 phage obtained by phage display, coated with silver nanoparticles. This hybrid phage-mediated killing of *F. nucleatum* led to enhanced immune response to the CRC and prolonged survival.^222^

Another potential phage application is phage-delivered programmable CRISPR systems that modulate pathogen functionality and abundance. Lam et al.^223^ demonstrated both *in vitro* and *in vivo* in colonized mice that treatment of an *E. coli* strain by the engineered filamentous M13 phage harboring CRISPR-Cas9 system is able to cause large chromosomal deletions in the targeted area and impaired bacterial growth. Phage λ was demonstrated as an efficient and precise delivery system for gene repression^224^ or engineering^225^ in *E. coli* both *in vitro and in vivo*. In another study, the *C. difficile*-infecting prophage was modified by introducing bacteria-targeting crRNA and removing lysogeny genes to reprogram the endogenous CRISPR-Cas system to cut the bacterial genome,^226^ thus aiding pathogen elimination.

Still, while phage therapy holds promise, there are still challenges in developing effective treatments. These include ensuring phage specificity, avoiding resistance development, and understanding the long-term impacts on the microbiome.

## Perspectives

**Figure 3.**
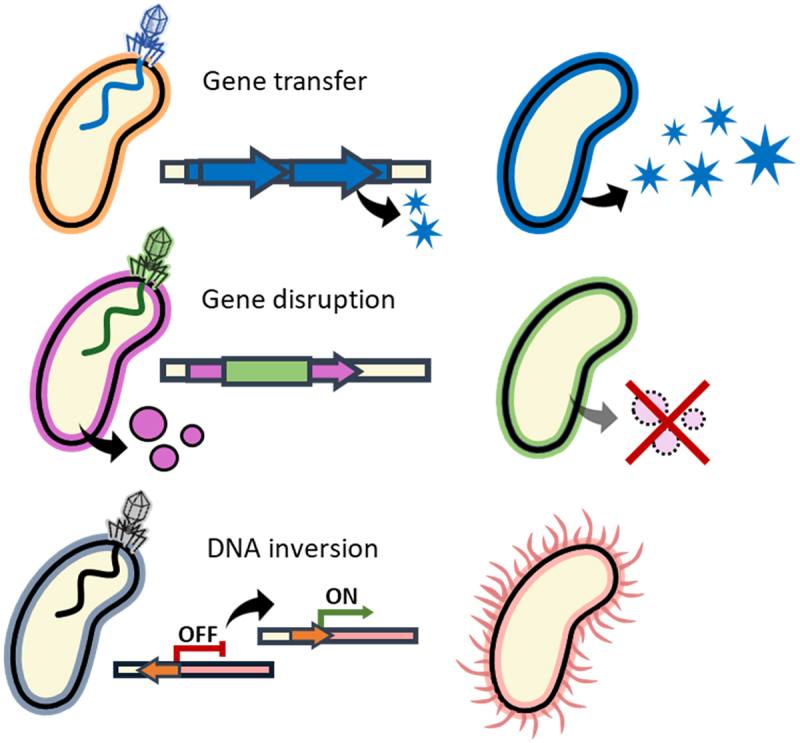
Phage-induced modulation of bacterial functionality.

The growing efforts to study the role of gut phages in microbiome functionality are promising. However, the mechanisms of phage-mediated regulation of bacterial metabolism (Figure 3) are still poorly characterized. Understanding the role of phages in both health and disease is particularly important for the medical applications of phages. Phage diversity in the gut microbiome remains largely unknown, and extensive work is required to explore gut phages and their hosts. The task is mainly complicated by the limitations of the culturing methods used for phage isolation and the prevalence of temperate lifestyle in the gut. Large-scale culturing approaches^227^ should be further developed in order to isolate gut phages and characterize their functionality.

Future research should also focus on the role of prophages in gut bacterial metabolism and functionality. This includes investigating the triggers for prophage induction, the impact of prophage-encoded genes on bacterial physiology, and the ecological consequences of prophage activity. By integrating this knowledge, we can develop a comprehensive understanding of prophages as modulators of the gut microbiota. Phage therapy approaches, which are currently focused on using bacteriophages to eliminate pathogens, can be significantly advanced by the use of prophages to confer advantageous traits to beneficial bacteria and suppress the harmful ones.

The role of phages in the gut microbiome extends beyond controlling bacterial composition and metabolism. Emerging research suggests that phages can interact with the mammalian immune system, potentially modulating immune responses and contributing to the maintenance of gut homeostasis.^171,172,175,183^ In turn, this could affect gut bacteria and have significant implications for health and disease. Research in this area can lead to new therapies for immune-related disorders, such as IBD, RA, and more. The potential impacts of phages on human cells and tissues are crucial to understand, and further work is needed to determine the application, safety, and efficacy of phage-based treatments.
